# Cryptic binding sites become accessible through surface reconstruction of the type I collagen fibril

**DOI:** 10.1038/s41598-018-34616-z

**Published:** 2018-11-09

**Authors:** Jie Zhu, Cody L. Hoop, David A. Case, Jean Baum

**Affiliations:** Department of Chemistry and Chemical Biology, Rutgers University, Piscataway, New Jersey 08854 USA

## Abstract

Collagen fibril interactions with cells and macromolecules in the extracellular matrix drive numerous cellular functions. Binding motifs for dozens of collagen-binding proteins have been determined on fully exposed collagen triple helical monomers. However, when the monomers are assembled into the functional collagen fibril, many binding motifs become inaccessible, and yet critical cellular processes occur. Here, we have developed an early stage atomic model of the smallest repeating unit of the type I collagen fibril at the fibril surface that provides a novel framework to address questions about these functionally necessary yet seemingly obstructed interactions. We use an integrative approach by combining molecular dynamics (MD) simulations with atomic force microscopy (AFM) experiments and show that reconstruction of the collagen monomers within the complex fibril play a critical role in collagen interactions. In particular, the fibril surface shows three major conformational changes, which allow cryptic binding sites, including an integrin motif involved in platelet aggregation, to be exposed. The observed dynamics and reconstruction of the fibril surface promote its role as a “smart fibril” to keep certain binding sites cryptic, and to allow accessibility of recognition domains when appropriate.

## Introduction

The extracellular matrix (ECM) in connective tissues contains a mixture of biological components that regulate cell migration, growth, and differentiation through cellular interactions. Making up 90% of all collagen in the human body, type I collagen forms large fibrillar structures that not only provide tensile strength to uphold tissue integrity, but also maintain biological functions through interactions with its many binding partners, including cell surface receptors, enzymes, and other ECM components^1–4^. For example, collagen interactions with integrin cellular receptors are important for platelet aggregation, cell development, differentiation, and hemostasis^5–7^. Collagen fibril degradation and turnover is dependent upon cleavage by matrix metalloproteinases (MMPs). Defects in collagen interactions are associated with fatal diseases, such as heart disease, cancer, and arthritis^8,9^.

**Figure 1:**
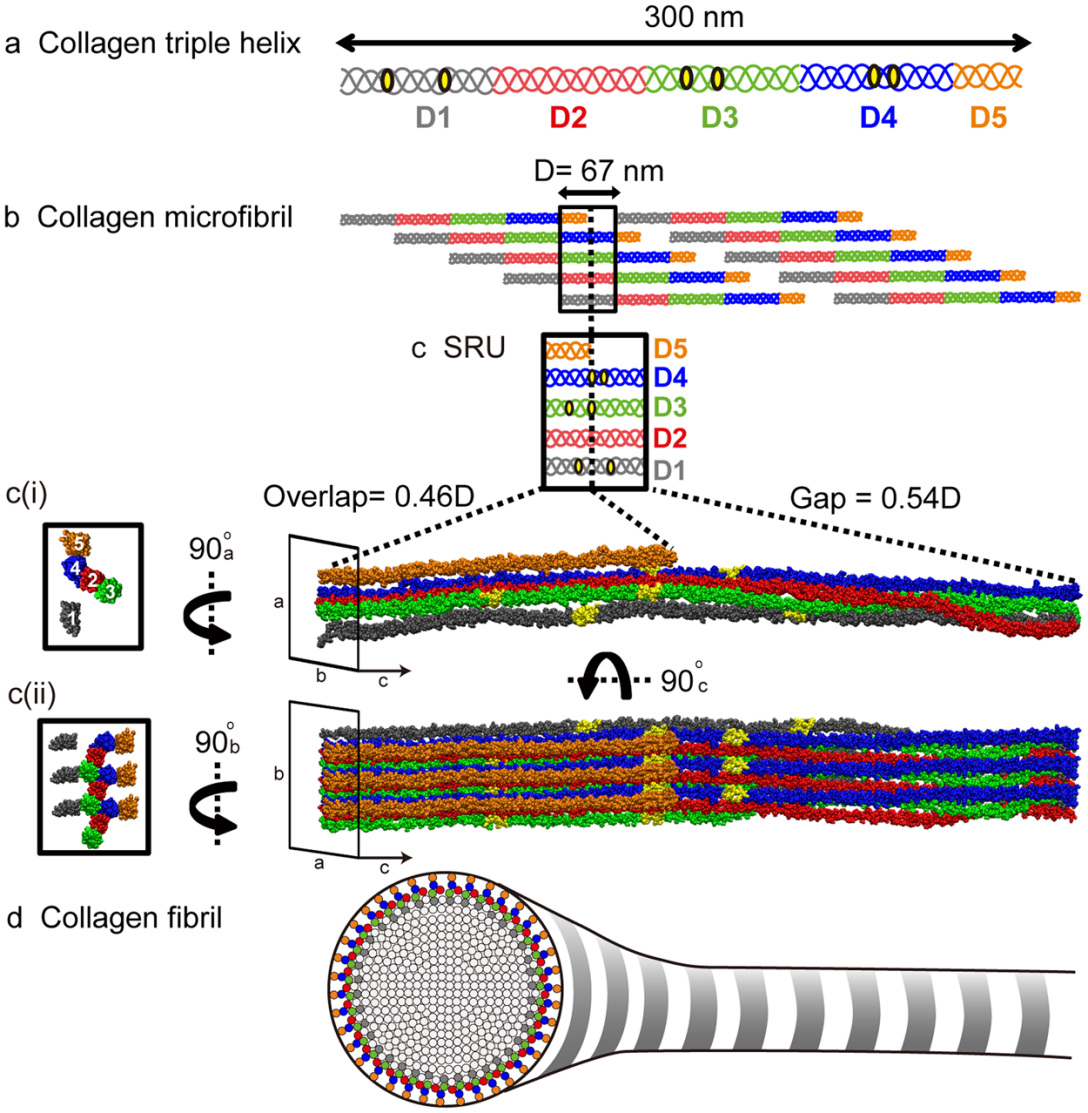
Type I Collagen structural hierarchy. (**a**) Collagen monomer: The type I collagen monomer is a heterotrimer triple helix consisting of two α1 and one α2 chains with approximate dimensions of 300 × 1.5 nm. The monomer is divided into five D-segments with D1–D4 having a length of 67 nm and D5 equal to 0.46D. (**b**) Microfibril: Five monomers pack in parallel and stagger by one D-period into microfibrils. Based on PDB: 3HR2^27^. (**c**) Smallest repeating unit (SRU): Isolating one D-period length of the microfibril gives the SRU, which contains the entire sequence of all five D-segments of the monomer in the configuration of the microfibril bundle. (ci) All-atom model of SRU rendered by VMD (http://www.ks.uiuc.edu/Research/vmd/)^66^. (cii) Three replicates of the SRU along the b-axis are created to define a representative fibril surface, shown in longitudinal view (right) and cross-section view (left). The short D5 divides the D-period into two regions; the “overlap” region contains segments D1 to D5, while the “gap” region only contains D1 to D4. In a and c, integrin binding motifs are indicated in yellow. (**d**) Fibril: The alternating overlap and gap regions create the characteristic “bright and dark” D-banding pattern viewed by electron microscopy when the collagen fibril is stained with heavy metal. The concentric packing of collagen monomers within a single fibril for the overlap region is viewed in the cross-section. Colored circles represent the estimated positions of collagen monomers on the surface layer and are color-coded by D-segments.

Interactions with full-length collagen monomers and fibrils are extremely challenging to study due to their huge size and complexity. Broad interaction domains on collagen monomers and fibrils have been identified through visualization of protein binding by atomic force microscopy (AFM) and electron microscopy (EM)^10–14^. More specific recognition sequences for dozens of type I collagen binding partners have been determined through elegant use of synthetic collagen mimetic peptides (CMPs)^15–17^ and recombinant bacterial expression systems that contain partial collagen sequences^18–20^. Through adhesion to triple helical CMPs, a minimal binding sequence for collagen-binding integrins has been established, GXX’GEX”, in which the Glu of the collagen motif coordinates a divalent metal cation with the metal ion-dependent adhesion site of the integrin inserted (αI) domain^21,22^. In the context of the linear triple helix, in which all possible binding sites are exposed (Fig. 1a), αI domains show preferential binding to a subset of these motifs^23^; high and moderate affinity binding motifs for α1I and α2I are colored yellow in Fig. 1. However, in the ECM, collagen monomers assemble into cylindrical D-banded fibrils via microfibrils^24,25^ (Fig. 1b–d). The bundling of monomers into the quasihexagonal arrangement^26,27^ buries many of these sites, making them unavailable for interaction (Fig. 1c). The approximate locations of the six highlighted integrin binding motifs are shown within the smallest repeating unit (SRU) of the fibril, which is one D-period length of the microfibril and contains a bundle of five unique segments from different collagen monomers (Fig. 1c). Collectively, these “D-segments” contain the entire type I collagen sequence. As the microfibrils assemble in all dimensions, forming a long cylindrical fibril superstructure with a circular cross-section of concentric layers^28^, only one face is left exposed for interaction (Fig. 1d). There are two possible models of the fibril surface; “surface A,” represented by D5 and D4 as shown in Fig. 1^29^, and “surface B,” represented by D1^30^ (see Fig. S1). Previous studies support the view that the surface proposed by Perumal *et al*.^29^ is a better fit of the corrugated profile of the type I collagen fibril from rat tail tendon observed by scanning electron microscopy and AFM^31,32^ and potential exposure of certain binding sites, such as those of decoron and MMPs^29,33–35^. Despite many of its binding motifs being obstructed, integrin α2β1 has been shown to indeed interact with mature type I collagen fibrils as visualized by immuno-EM imaging and mediation of cell spreading, yet through undetermined binding sites^36^. Remarkably, although critical binding sites are buried inside the fibril, these and numerous other cellular processes reliant on collagen–protein interactions are accomplished. It has been suggested that specific packing of triple helical monomers within the fibril makes important protein-binding sites cryptic to become available only in specific instances^34,35^. However, how partner binding sites become available in the supermolecular fibril assembly is not understood.

Here we use an integrative approach that combines all-atom molecular dynamics (MD) simulations with AFM of type I collagen fibrils to characterize the interaction surface of the type I collagen fibril from rat tail tendon. Through MD, we probe the dynamics and surface reconstruction of the surface layer of the type I collagen fibril from a starting model derived from the X-ray fiber diffraction model of the type I collagen fibril unit cell from rat tail tendon^27^. Although the X-ray fiber diffraction model provides only the Cα positions and does not have the resolution to make conclusions about atomic-level details of triple helical conformations within the fibril, it importantly provides the arrangement of collagen monomers within the repeating unit of the fibril, which allows us to model relative positions of interaction sites near the fibril surface. High resolution AFM experiments give nanoscale insight into the surface structure. Important contributions from X-ray diffraction and microscopy provide static snapshots of a supermolecular assembly, but dynamics, especially at the binding interface, which are potentially critical for cellular function, have not been investigated and are inaccessible by these methods. Previous computational studies of infinite periodic models of collagen fibrils provide structural and mechanical information of the fibril core^37–40^, but do not distinguish the interaction surface of the fibril from the interior. We have performed an all-atom MD simulation of an early stage, fully solvated type I collagen fibril model with an explicit interaction surface that allows sampling of rare conformational events on the surface. In our model, we used a matrix of SRUs to represent the fibril surface, since the surface is identical around the cylindrical fibril superstructure^28^ (Fig. 1d). Through the MD simulation, we observe that the fibril surface is not merely a rigid rod, but exhibits large fluctuations and displacements of particular segments within the D-period. The reconstructed fibril results in the inward contraction of the gap region and the outward expansion of the overlap region creating an overall denser packing of monomers in the surface layer and exposing certain previously hidden interaction sites. The conformational fluctuations change the accessibility of certain binding regions over time and suggest that the dynamics on the surface are critical for collagen fibril interactions and dependent cellular processes.

## Results

### The type I collagen interaction surface undergoes conformational fluctuations on the nanosecond timescale

**Figure 2:**
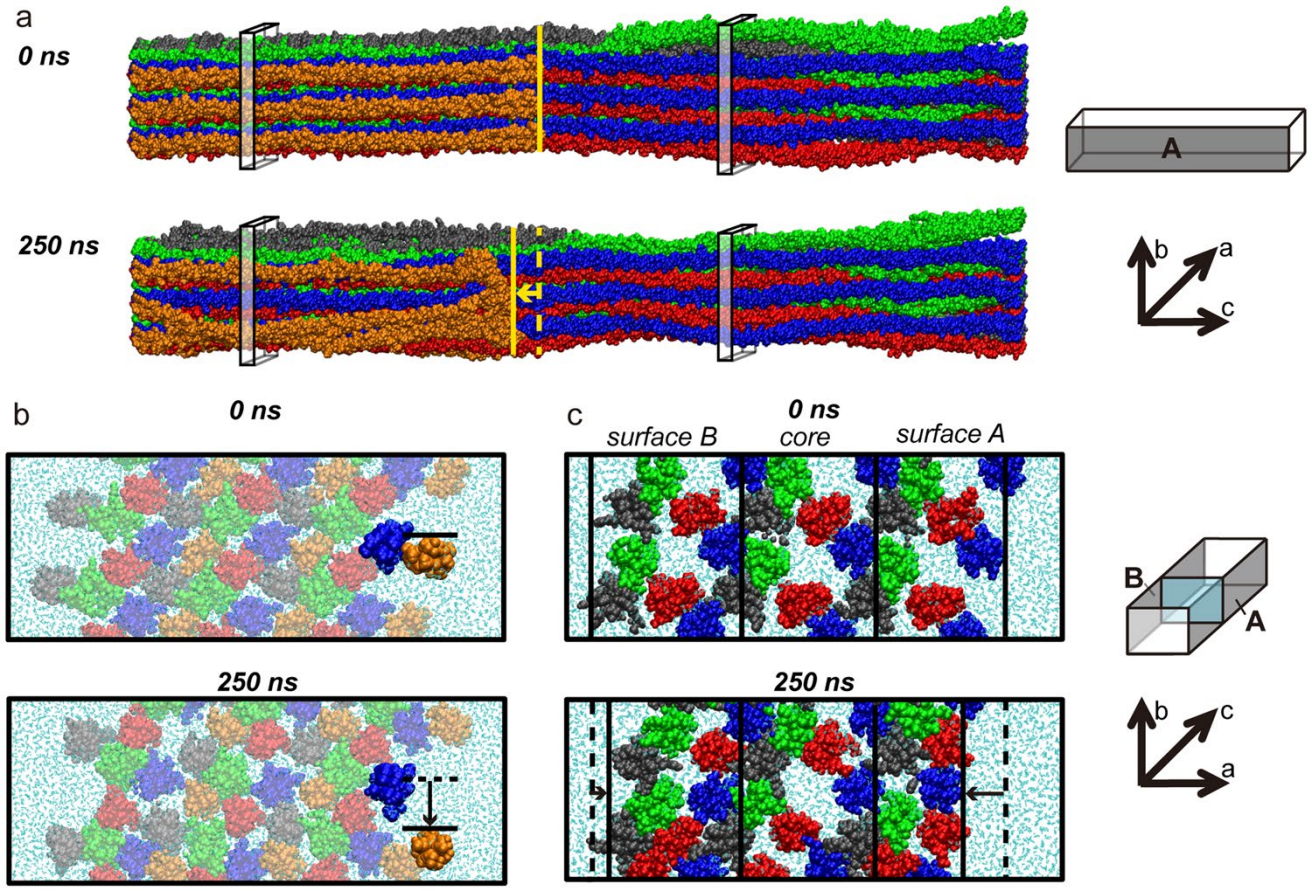
Three major movements observed during the 250-ns MD simulation. Snapshots from the starting (0 ns) and ending (250 ns) time points are shown. D-segments are color-coded: D1- gray, D2- red, D3- green, D4- blue, and D5- orange. (**a**) Longitudinal view of the full D-period model. The C-telopeptide on the D5-segment shifts N-terminally, exposing sites on D4 previously hidden by the C-telopeptide. The edge of D5 is indicated by the solid yellow line in both snapshots. The dashed line at 250 ns demarcates the edge of D5 at 0 ns. The four transparent slices are shown as cross-section view in panels b and c. (**b**) Cross-sectional views of slices taken at 10–12 nm from the N-terminus along the c-axis. The downward displacement of D5 from its starting position is indicated by black lines in the same manner of those in panel a. Motions in the middle of D5 open a cavity that allows access to D4. (**c**) Cross-sectional views of slices taken at 44–46 nm from the N-terminus along the longitudinal axis in the gap region. The surface layer of the gap region contracts inward toward the core, exposing the originally partially hidden D2 and expelling waters (cyan) from within the surface layer. Solid black lines demarcate boundaries of the surface layers and the core layer at the time point indicated. Dashed black lines show the original position of the layer boundary. Boxes on the right of panels a, b and c indicate the orientation of the 3a3b fibril model, with the gray sides representing the surfaces and the blue plane representing a cross-section slice.

We monitored the motions of the collagen monomers at the fibril surface during MD simulations to characterize the surface reconstruction. MD simulations were performed on a model that contains three copies of the SRU along the a-axis and three copies along the b-axis, the “3a3b” model. The three layers along the a-axis are surface layers A and B that each have one face exposed and the core layer, sandwiched between the surface layers, which represents the fibril interior. In this way, one SRU in each of the three layers is surrounded by all of its neighbors as in the full fibril. All analyses are presented for surface A, which places D4 and D5 on the immediate interaction surface. The alternative surface B places D1 at the fibril forefront. From this starting structure (0 ns) to the end point (250 ns) of our MD simulation, three major conformational changes are apparent (Fig. 2): (1) longitudinal translation of the C-telopeptide, (2) downward displacement of D5 in the overlap region, and (3) contraction of the surface layer in the gap region. From the longitudinal view (Fig. 2a), we observe a displacement of the C-telopeptide in D5 in the N-terminal direction. This shift exposes a patch of the D4-segment (Fig. 2a, blue) that was previously occluded by the C-telopeptide. The movement also allows the D5 segment to shift, which opens a wider range of motion for residues in the middle of the segment. In some of the MD frames, the middle of D5 is observed to fluctuate along the a-axis, bulging away from the surface and returning back (Fig. S2). The cross-section view of a slice from the middle of the overlap region (Fig. 2b) shows that the D5-segment also has downward movement along the b-axis, which creates a cavity, exposing much of the overlap region of D4 (blue). While fluctuations in the D5 cause outward expansion from the surface, the gap region of the surface layer contracts inward toward the fibril core, creating a much denser packing of monomers in the surface layer and exposing the previously hidden D2-segment (Fig. 2c, red). This denser packing expels water from the surface layer (Fig. 2, cyan). In order to characterize these motions in further detail over the time course of the simulation and address how the surface reconstruction may facilitate ligand binding, we analyzed time points of the simulation in terms of displacements, dynamics, hydrogen bond modulation, and accessibilities.

### Internal dynamics of the fibril surface contribute to movements of outermost monomers

**Figure 3:**
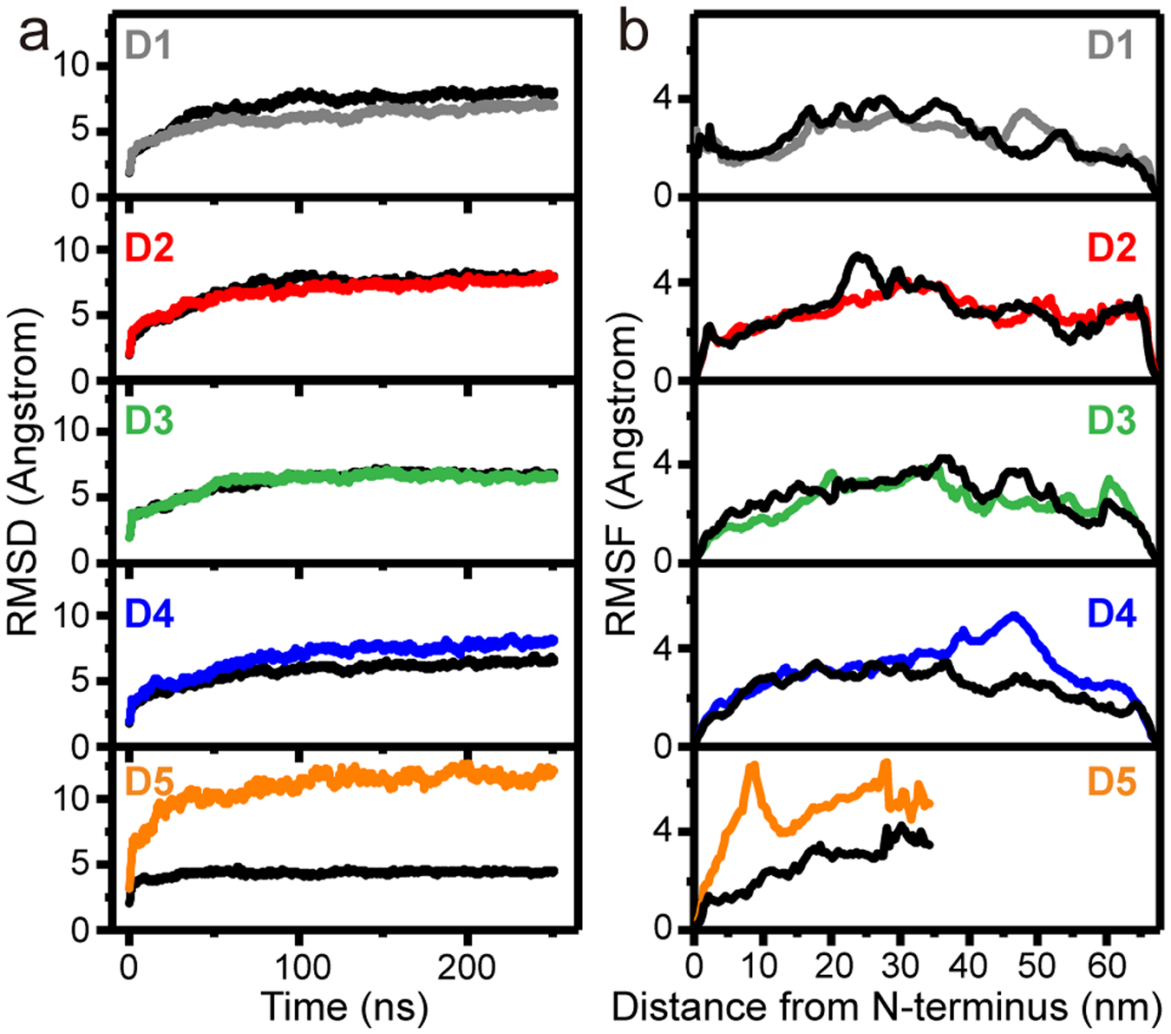
Internal motions within the fibril model. (**a**) Root-mean-square deviation (RMSD) during the 250-ns MD simulation of D-segments 1–5 within the core layer (black) and surface layer (color-coded as in Figs 1,2). (**b**) Root-mean-square fluctuation (RMSF) of D-segments 1–5 over the time of the simulation. RMSF of surface layer (colored) and core layer (black) are overlaid and aligned by distance from the N-terminus in the SRU.

The internal motions of the fibril model are characterized by the root mean square deviations (RMSD) and root mean square fluctuations (RMSF) over the course of the MD simulation (Fig. 3). To distinguish motions of the interaction surface, we have analyzed the surface and the core layers separately. We consider only the middle microfibril bundle along the b-axis since it is surrounded by its neighbors on all sides. In comparison of the RMSDs and RMSFs of the core and the surface layer, differences between the layers are interpreted as distinct motion in the surface layer relative to the interior of the fibril.

The RMSD is a measure of the distance of atoms from the starting position. In Fig. 3a, we plot RMSD vs. simulation time of the indicated D-segment in the core layer (black) and the same segment on the surface (color). The RMSDs of all segments in the core and surface layers roughly converge within about 100 ns. We observe the largest deviation in RMSD between the layers in D5 (orange), the frontline of the fibril surface. While the core layer is displaced only by ≈4 Å by the end of the simulation, the surface layer has a much greater displacement of ≈12 Å. This especially high RMSD of D5 in the surface layer is in agreement with the large movements in all directions of D5 captured in the snapshots in Fig. 2. The D4 (blue) segment also shows a slightly higher RMSD (by ≈2 Å) at the surface than within the fibril core. In contrast, segments D2 and D3 do not deviate in RMSD on the surface compared to the core, and D1, the innermost D-segment from this surface, actually has a marginally lower RMSD on the surface. It should be noted that we put restraints on both termini of each D-segment, except for the N- and C-telopeptides, to mimic the covalent bonding to N- and C-terminal residues that are part of the same monomer in an adjacent D-period. These restraints introduce bias by limiting the motions of D-segments at the termini. For instance, in full collagen fibrils, the D4 C-terminus would be covalently bound to a D5 segment in the subsequent D-period. Given that D5 shows high RMSD, its displacement could influence the bound D4 and enhance the D4 RMSD beyond what we have calculated here. These results suggest that the most substantial conformational changes to the fibril surface occur in the outermost two triple helices on the surface, D4 and D5 (Fig. 3a).

While the RMSD is informative of the displacement of a particular region over time, the RMSF is a measure of the fluctuations of each residue during the entire MD simulation. To quantitatively characterize the most dynamic areas within the collagen fibril, we performed RMSF calculations after the system was equilibrated (Fig. 3b) and compared the surface and core layers as in the RMSD analysis. The highest fluctuations in the surface layer relative to the core are again in D5 and in the gap region of D4, on the forefront of the interaction surface (Fig. 3b). Given the extensive flexibility of the majority of the D5 segment, in the absence of restraints, the RMSF of its N-terminus is not likely to be static as suggested in Fig. 3b. Instead, its dynamics could extend to the covalently bound D4 segment in the adjacent SRU, and potentially add to the fluctuations in the D4 C-terminus. No considerable differences in fluctuations are observed in the D1, D2, and D3 relative to the core layer. Together, the trends in RMSD and RMSF indicate that regions closest to the fibril surface are more dynamic and show sizeable movement over the course of the simulation compared to the core of the fibril.

### Formation of protein–protein H-bonds supports tighter monomer packing in the surface reconstruction

**Figure 4:**
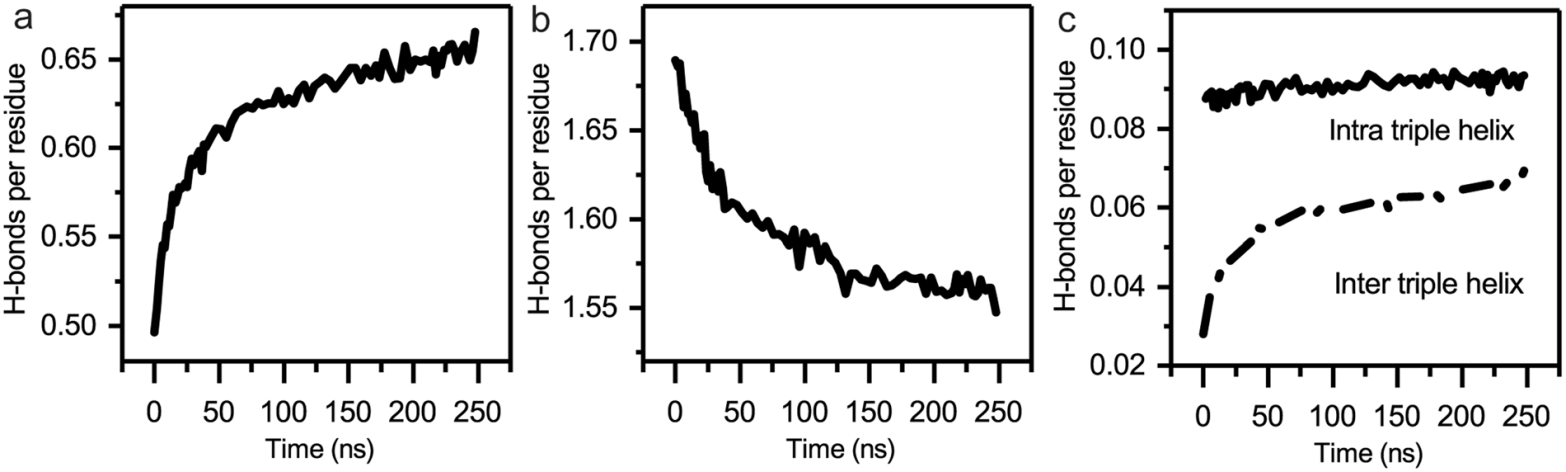
Hydrogen bond (H-bond) modulations in the MD simulation. (**a**) Protein–protein and (**b**) protein–water H-bonds per residue in the fully solvated collagen fibril model through the MD simulations. (**c**) Buildup of side chain involved intra- (solid) and inter- (dashed) triple helix protein–protein H-bonds per residue.

The formation of hydrogen bonds (H-bonds) is an important factor in protein folding and protein–protein interactions. By monitoring the number of protein–protein H-bonds with respect to time in the whole collagen fibril model, we observe that the protein–protein H-bonds increase by 0.169 H-bonds per residue, and protein–water H-bonds decrease by 0.142 H-bonds per residue within 250 ns of MD simulation (Fig. 4a,b). The gain of protein–protein H-bonds with loss of protein–water H-bonds is consistent with increased monomer packing in the fibril. To determine the contribution of H-bond buildup due to monomer packing, we focused on trends of H-bonding between triple helices. Backbone atoms of different triple helices are distant enough that inter-triple helix backbone–backbone H-bonding is rare throughout the time course of the simulation (Fig. S3). The build-up of the inter-triple helix sidechain-involved H-bonds, however, is indicative of the supermolecular packing. These H-bonds continue to increase during the entirety of the MD simulation (Fig. 4c), not converging within 250 ns. At the same time, sidechain-involved H-bonds within triple helices remain constant. This formation of inter-triple helix protein–protein H-bonds with breakage of protein–water H-bonds supports tightening of the monomer packing within the model. Other factors, such as exposure of hydrophobic regions and distribution of charges on the fibril surface may also accompany the reconstruction of the fibril surface.

### Experimental AFM images topographic features of the type I collagen fibril surface

**Figure 5:**
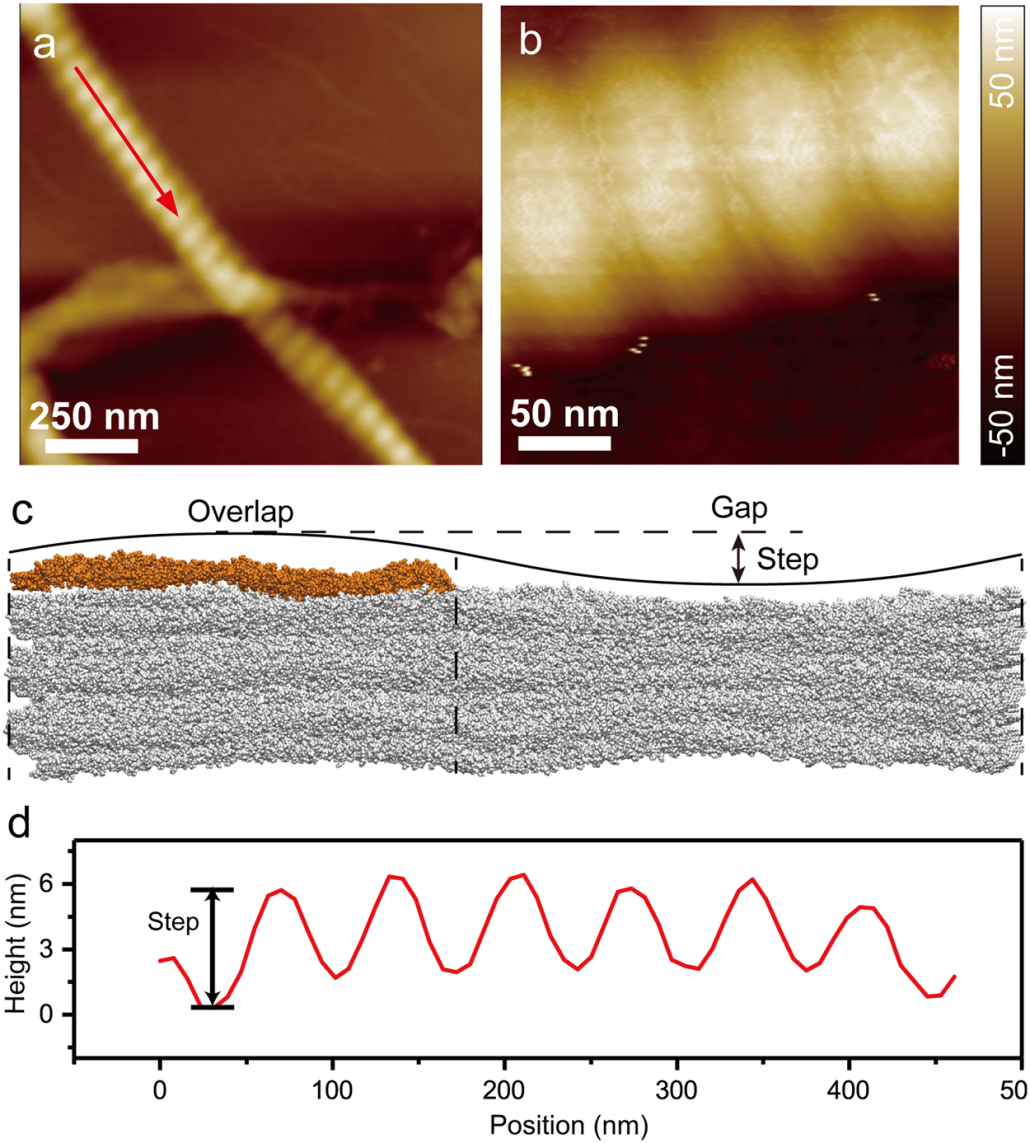
Measuring height difference between overlap and gap regions. (**a**,**b**) AFM height images of type I collagen fibrils with dimensions of (**a**) 2 µm × 2 µm and (**b**) 250 nm × 250 nm. (**c**) Schematic describing how the AFM height profile relates to the overlap and gap regions of the collagen fibril. D5 on the surface in the overlap region is colored orange. The step-height is the height difference between the peak of the overlap and the valley of the gap. (**d**) The height profile taken along the red arrow in (**a**). The height profile has a periodicity ≈67 nm, consistent with the D-period of the collagen fibril model.

The MD simulation shows reconstruction of the type I collagen fibril that would result in changes to the surface topography. For example, the inward contraction of the gap and the outward expansion of the overlap region increase the depth between the highest point in the overlap region and lowest point in the gap region (Figs 2 and S2). These topographical features could be measured by AFM, which is an excellent tool to specifically probe physical and mechanical properties of the surfaces of materials and proteins. We imaged several type I collagen fibrils from reconstituted rat tail tendons in air adsorbed to mica. By analyzing the height profiles of the D-bands of an isolated fibril, we measured an average height difference between the overlap (peak) and the gap (valley) regions to be 4.1 ± 0.4 nm (Fig. 5). The overall height and the relative heights of the overlap and gap regions are sensitive to environmental conditions^41,42^. Hydration induces swelling of collagen fibrils and may influence this step-height.

### Type I collagen fibril reconstruction has implications in the accessibility of ligand binding sites on the fibril surface

Cell receptor and ligand binding sites on collagens have largely been determined on triple helical peptides, recombinant bacterial collagen constructs, and from imaging proteins binding to collagen monomers by microscopy methods^10–17^. In its monomeric form, all possible binding sites are exposed and available for interaction. However, when bundled into the supermolecular fibril, several of these binding sites become hidden from the interaction surface. To characterize accessibilities of binding motifs in the collagen fibril, we calculated solvent accessible surface area (SASA) around each residue of the fibril model, excluding surfaces on the interior of the fibril, i.e. those not accessible from the fibril interaction surface. To eliminate SASA of the interior, we used a spherical probe approximately one-half the size of the cavities within the gap region (with a radius of 8 Å). This probe size is still small enough to be sensitive to small deviations in SASA across the surface. Comparing the 8.0 Å SASA of residues in the surface layer of surface A in the starting model and at the end of the simulation, we find that fluctuations on the fibril surface substantially enhance accessibilities in parts of the fibril that are buried prior to reconstruction.

**Figure 6:**
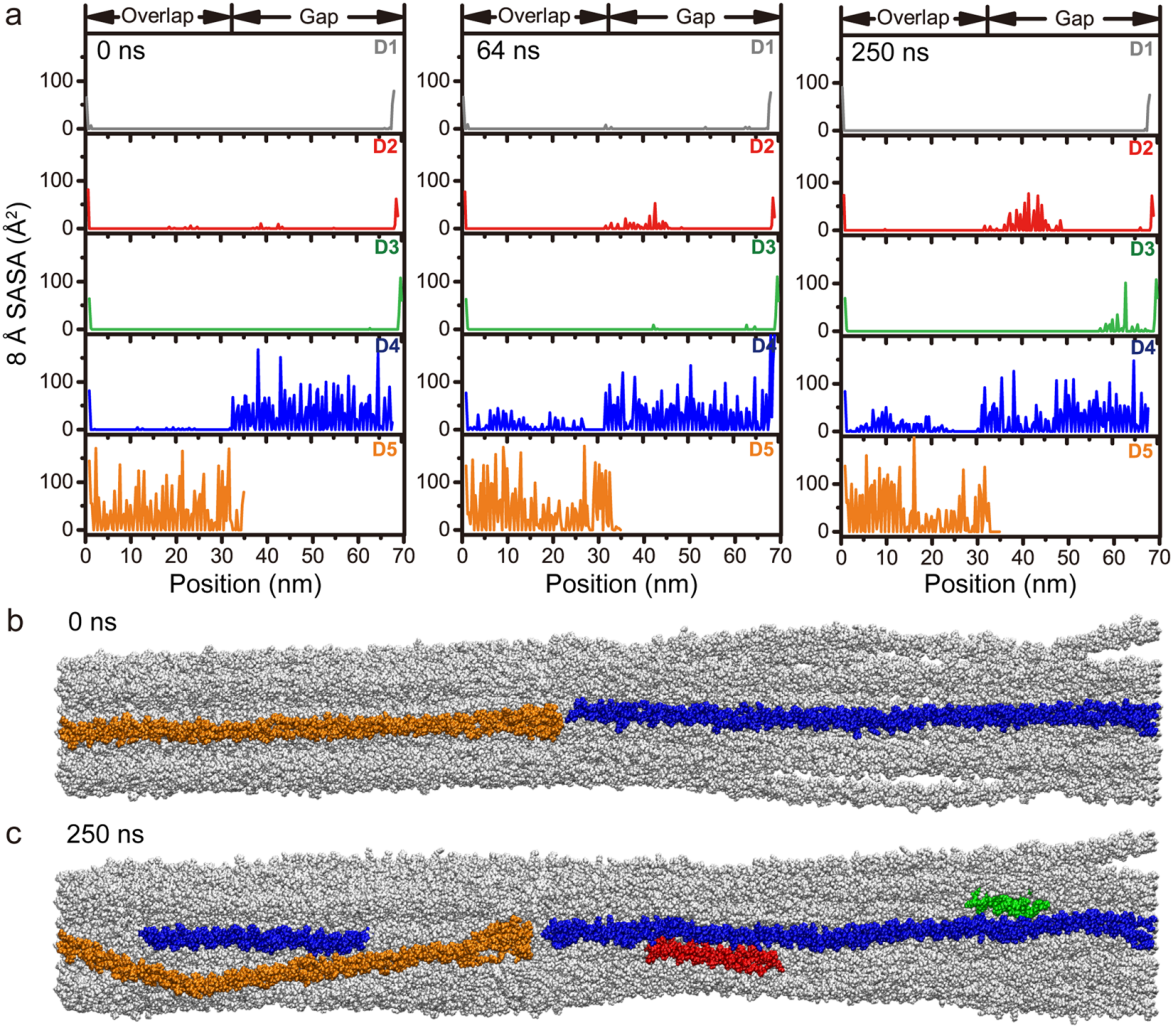
8.0Å SASA of surface A of the fibril model at 0 ns, 64 ns, and 250 ns time points of the MD simulation (**a**). (**b**,**c**) Longitudinal view of the fibril model highlighting residues with 8.0 Å SASA higher than 15 Å^2^ in (**b**) the starting model and (**c**) at 250 ns. Although invisible in the starting model, D4 (blue) in the overlap region and parts of D2 (red) and D3 (green) in the gap region become accessible due to fibril surface reconstruction.

The modulation of the 8.0 Å SASA of surface A due to motions in the dynamic surface is shown for three time points in the simulation (Fig. 6a). In the starting structure, the 8.0 Å SASA was ≈0 Å^2^ on the entire lengths of D1, D2 and D3 and in the overlap region of D4 (Fig. 6a). Conversely, residues directly on the binding surface (D4 in the gap region and D5 in the overlap region) have considerably high 8.0 Å SASA since they are completely exposed. Consistent with the turn of the triple helix, even in the regions of high 8.0 Å SASA, the accessibilities drop to zero every three residues since the glycines of the (G-X-X’)_n_ repeating sequence point to the center of the triple helices and are not accessible from the interaction surface. Throughout the simulation, specific regions in D2 (30–50 nm) and D3 (57–65 nm) show substantial increase in SASA over time (Fig. 6). D4 in the overlap region has fluctuating accessibilities over time, which is likely influenced by the dynamics of D5. Additionally, the N-terminal displacement of the C-telopeptide extends the accessible region on D4 at the junction of the gap and the overlap regions. Modulations in accessibility over time may provide a key as to how reconstruction of the type I collagen fibril surface may facilitate ligand binding.

**Figure 7:**
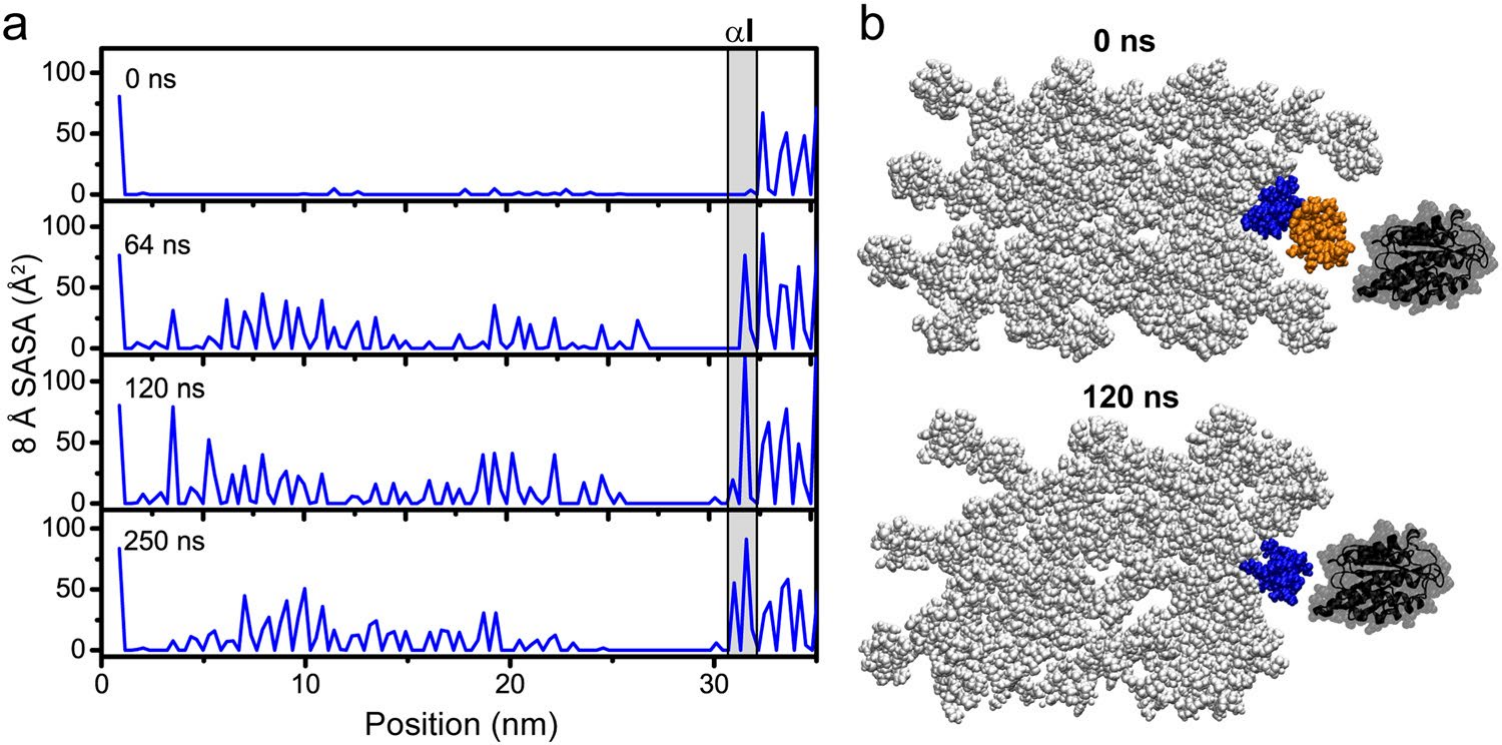
Measuring accessibility of a major ligand binding region on the D4-segment that contains an integrin αI domain binding site. (**a**) 8.0 Å SASA at 0 ns, 64 ns, 120 ns, and 250 ns MD simulation time of the overlap region of D4. The integrin αI domain binding site, GQRGER, is highlighted by the gray box. (**b**) Cross-sectional view of the GQRGER αI domain binding site on D4 (gray box in panel a), nine residues deep along the longitudinal axis, at 0 ns (starting model) and 120 ns (maximal accessibility of this site). GQRGER on the D4-segment is blue and the neighboring C-telopeptide on the D5-segment is orange. The integrin α2I domain is shown in black (PDB ID: 1aox^69^). In the starting structure, the C-telopeptide immediately on the fibril surface is obstructing αI access to GQRGER. However, after 120 ns, the C-telopeptide is translated longitudinally, and out of the cross-sectional slice, allowing αI access to the GQRGER binding motif in the fibril.

Through analysis of these SASA calculations, we find that fluctuations of the fibril surface reveal cryptic regions important for ligand interaction. In the overlap region, dynamics of the fibril surface open access to at least one of the hidden major ligand binding regions on D4^3,4^ (Fig. 7a). This is a concentrated zone of molecular binding partners, including MMPs, collagen-binding integrins, discoidin domain receptors, heat shock protein 47, and fibronectin^3,4^. In this region, the SASA is not only seen to increase with longer simulation time, but has fluctuating accessibilities over time, especially in the region between 18–28 nm. We specifically probed an integrin αI domain binding site within this region at the edge of the overlap with the sequence GQRGER (Fig. 7). In the starting model, this site is hidden by the C-telopeptide in D5 and has an 8.0 Å SASA value of ≈0 Å^2^, and as a static structure, it would seem to be unavailable for binding. Monitoring the 8.0 Å SASA over time, we observe variability of accessibility at this site. By 120 ns, the binding site has maximum accessibility (Fig. 7a). At this point, the C-telopeptide has translated longitudinally toward the N-terminus and unveils the integrin αI domain binding site, as shown in the cross-sectional view of Fig. 7b. Although more open, dynamics within the fibril cause the 8.0 Å SASA to fluctuate, as seen by the lower accessibility at 250 ns (Fig. 7a). Variable accessibility of specific regions of the fibril surface through time is consistent with a dynamic surface that is flexible to assume a multitude of conformations.

## Discussion

In this report, we present an early stage atomic model of the type I collagen fibril surface. This is the first all-atom MD simulation of a collagen fibril model that allows discrimination of the binding surface from the interior. Although our simulation is certainly not fully equilibrated (and hence the description of surface reconstruction is incomplete), we have found that fluctuations at the interaction surface of the type I collagen fibril allow sampling of rare events on the hundreds of nanoseconds timescale. These fluctuations involve three major movements (Fig. 2): (1) downward movement of D5, (2) inward contraction of the gap region, and (3) longitudinal displacement toward the N-terminus of the C-telopeptide. We analyzed the RMSD, RMSF, and H-bond perturbations throughout the 250 ns MD simulation. The RMSD and RMSF show that the outermost monomers on the surface of the fibril have greater spatial deviation from the starting structure and are more flexible than analogous regions in the fibril interior (Fig. 3). We find that the fluctuations are accompanied by formation of new protein–protein H-bonds and disruption of protein–water H-bonds (Fig. 4a,b). The number of protein–protein H-bonds on the surface increases over the timeframe of the MD simulation replacing protein–water H-bonds and expelling water from the surface in the process. This is similar to the collagen fibrillogenesis process, which is driven by the loss of water molecules from the protein surface^43–46^. The formation of protein–protein H-bonds optimizes supermolecular packing of monomers, as we observe an increase in sidechain–sidechain inter-triple helix hydrogen bonds (Fig. 4c). The quantification of the H-bonding is consistent with our observation that the surface layer of the gap region becomes more compact, tightening monomer interactions while displacing water in the surface reconstruction. This difference in monomer packing between the fibril surface and interior is consistent with an inhomogeneous assembly proposed by several researchers^47–50^. In particular, Gutsmann *et al*. suggested that the collagen fibril has a harder, denser surface layer and softer core^48^. They proposed that this inhomogeneous structure might be more resistant to bending and reduce deformation of collagen fibrils.

The reconstruction changes the surface topography of the fibril. AFM allows us to experimentally characterize the fibril surface in terms of height profiles, electrostatics, and mechanical properties; and it provides a complimentary tool to probe the MD fibril surface model. It should be noted however, that these computational and experimental methods provide information on different timescales, on the order of hundreds of nanoseconds in MD and seconds in AFM. At this stage, we have measured the overlap–gap step-height on the surface to be 4.1 ± 0.4 nm, as a starting point. It is interesting to note that this step-height is somewhat larger than would be expected based on the starting model for the MD simulation. Further work is being pursued to better understand the relationship between the MD simulations and AFM measurements. The topography of the collagen fibril is sensitive to environmental conditions, such as hydration, pH, and salt concentrations. Soft, biological materials can now be imaged to sub-nanometer resolution in physiological buffers^51–53^. Current studies in our laboratory are working toward characterizing the collagen surface topography and mechanical properties in different environmental conditions. More recently, the evolution of high speed AFM technology has provided a way to study protein dynamics on millisecond timescales^54,55^. With these advancements, future studies will allow us to address biologically interesting questions about physical, electrical, mechanical, and dynamical properties of the collagen fibril surface.

We present the analysis of our MD simulation assuming that segments D4 and D5 are exposed to the surface. The exposure of this surface is based on fitting the type I collagen microfibril X-ray diffraction model to the corrugated profile of the fibril observed by SEM and AFM^31,32^ and access to certain ligand binding sites, especially those of decoron and MMPs^29,33–35^. We would expect that in order for ligand binding to occur, the binding site should be accessible from the binding surface^29,34^. However, this does not necessitate that the binding site is open at all times given the now observed dynamics of the fibril surface. In our time-dependent SASA analysis, we find that cryptic sites that are unavailable in the static structure, become exposed due to the observed conformational fluctuations of the type I collagen fibril. As shown in the 8.0 Å SASA (Fig. 6), the flexibility of D5 allows access to parts of D4 in the overlap region, and conformational changes in the gap region expose parts of D2 and D3. The ability of the collagen fibril surface to sample these conformations, and not maintain a single, rigid conformation may enable or inhibit cellular processes through exposure of cryptic sites, such as those in the major ligand binding region in the overlap of D4, which houses binding sites for MMPs, secreted protein acidic and rich in cysteine (SPARC), discoidin domain receptor 2 (DDR2), phosphophoryn, fibronectin, and integrins. In this way, these ligand binding sites are cryptic, and the dynamics of the monomer on the collagen fibril surface provide a means of exposure. Additionally, previous MD simulations have shown that dynamics of the individual chains within the triple helices themselves play an important role in facilitating binding processes^56,57^.

Our early model of the type I collagen fibril surface provides a new framework upon which future studies can build and that now allows us to address important biological questions, such as: What regulates the conformational transitions that provide access to cryptic binding sites? How are the surface dynamics perturbed by environmental conditions, such as pH and salt concentration? Are there slower timescale motions that may affect ligand binding? How might the reorganizations that occur on this timescale affect biological activity? Our simulation considers the surface layer of an isolated fibril. Further studies on the influence of environmental conditions or the impact of interacting molecules on the fibril dynamics may help to gain a better understanding of the regulation of these dynamic processes. Our current study is limited to fast, nanosecond timescales accessible by MD. From this, we cannot deduce events that occur on longer timescales. However time resolved AFM may be instrumental in observing collagen fibril surface rearrangements and ligand binding in action.

Collagen fibrils are often presented as long, rigid rods that provide tensile strength to connective tissues, such as bones and tendons. However, within the ECM, they are also very biologically active, interacting with numerous cell receptors, enzymes, and ECM components to carry out critical cellular functions. Here, we ask how collagen binding partners are able to access their recognition motifs that are seemingly sequestered by the complex collagen fibril architecture of bundled triple helices. MD and AFM are excellent techniques by which we can characterize the collagen fibril surface. Through an all-atom MD simulation of a type I collagen fibril surface model, we show that the fibril surface is not merely a rigid rod, but is actually dynamic on the nanosecond timescale and samples conformations not observed in static models. Through reconstruction of the fibril surface, cryptic binding sites are unveiled for several collagen binding partners. Fluctuations of the C-telopeptide and D5 especially expose the major ligand binding region of D4, including an integrin αI domain binding motif, GQRGER. The observed dynamics and reconstruction of the fibril surface promote its role as a “smart fibril” to keep certain binding sites cryptic, and to allow accessibility of recognition domains when appropriate. This suggests that through the transient availability of binding sites, collagen binding partners are able to interact with the collagen fibril to uphold their cellular functions. In addition, alternate conformations at the fibril surface expand possible drug targets against fatal collagen diseases.

## Methods

### Constructing and solvating the all-atom collagen fibril periodic model

We created the all-atom starting structure by combining high resolution models of crystallized collagen-like peptides and the low resolution X-ray fiber diffraction model of the type I collagen fibril from rat tail tendons, adapting the methodologies from de Leeuw *et al*.^37^. We used THeBuScr^58^ and Scwrl^59^ programs to predict the all-atom model of the triple helical domain of type I collagen based on the sequence translated from genes COL1A1 (P02452) and COL1A2 (P08123) in the UniProt Knowledgebase (www.uniprot.org). This creates a perfectly straight triple helix without supermolecular structure. The Cα atoms of the N- and C-telopeptides were added to the straight triple helix model based on the X-ray fiber diffraction structure (PDB ID: 3HR2)^27^ and all other atoms were added by the program LEaP in the AMBER package^60^. The all-atom model was then fit to the supermolecular structure of PDB entry 3HR2 by a best-fit rotation and translation of the Cα atomic coordinates. In vacuum minimizations were carried out to remove bad contacts. We used the AddToBox utility in the AMBER package, which is designed for crystal simulations, to add 11980 explicit water molecules to our system based on de Leeuw’s trial and error result^37^. We performed 15,000 steps of minimizations and 100 ps of heating from 0 K to 310 K, followed by production with gradually decreasing restraints from 10 to 0.1 kcal/mol × Å^2^ applied to all protein atoms.

### Constructing the collagen fibril surface model

The X-ray fiber diffraction structure of the three-dimensional arrangement of collagen molecules in naturally occurring type I fibrils from rat tail tendon (PDB ID: 3HR2)^27^ determined that the SRU of the collagen fibril contains all five D-segments from successive collagen monomers. The SRU unit cell is 678 Å long, 27 Å wide and 40 Å deep. We built an all-atom model of a single SRU by truncating the last frame of the all-atom periodic collagen model MD simulation every one D-period and packing them into a single unit cell. The five D-segments are indicated by colors in Fig. 1. The 3a3b collagen fibril model was built to be one unit cell long, three unit cells wide and three unit cells deep, containing two possible surfaces: surface A and surface B (Fig. S1). The other two boundaries (a–c plane) are not fibril surfaces since full-length fibrils keep expanding in the b dimension. We built only three units along the b axis to represent the periodically extending surface to minimize the computing expense. The 3a3b model was neutralized by Cl^−^ ions and solvated as a solute in a 12 Å buffer of explicit TIP3P water molecules by the program tLEaP in the AMBER package^60^.

### Molecular dynamics (MD) simulation

All MD simulations were performed in the AMBER2017 package^60^. The protein was treated with the ff14SB force field^61^, and the solvent water molecules were described using the TIP3P model^62^. A minimization was first carried out to remove bad contacts in the initial structure. The system was then gradually heated up to 300 K for 100 ps in the NVT ensemble using the Berendsen thermostat^63^. Equilibration in NPT ensemble was then performed with gradually decreasing weak restraints from 5.0 to 0.5 kcal/mol × Å^2^ applied on all protein atoms until the density of the system reached 1.0 g/mL. Finally, the production run was carried out for 250 ns in the NVT ensemble with 2.0 kcal/mol × Å^2^ restraints on three residues of the N- and C-termini of each D-segment except for the N- and C- telopeptides. These restraints were used to maintain the interactions between D-segments in adjacent D-periods in full-length collagen fibrils. The SHAKE algorithm was used to constrain all bonds involving hydrogen atoms during the simulations^64^. Hydrogen masses were repartitioned onto bonded heavy atoms using the algorithm by Hopkins *et al*.^65^, which allowed a long integration time step of 4 fs to be used to accelerate the simulations.

### Analysis of simulation results

The trajectories were visualized using the VMD software^66^, in which the mapping of collagen receptor binding motifs were performed by changing colors of their indexes. Three microfibrils in the 3a3b model were selected to represent either surface A, surface B or the core layer (Figs 2c and S1) because they are surrounded by all the neighbors present in full length collagen fibrils. The cpptraj utility in the AMBER package^67^ was used to perform RMSD, RMSF and hydrogen bonding analyses with default settings.

### Solvent Accessible Surface Area (SASA)

The Molecular Surface (MS) program^68^ was adopted to perform solvent accessible surface area (SASA) calculations, which give the surface area of individual residues that can be approached by a probe of an indicated radius. The default probe size is 1.4 Å, which is the radius of a water molecule. We used a larger 8.0 Å probe size to characterize the accessibilities of collagen binding partners. The SASA values of residues on the same position of the three chains within a triple helix were averaged and plotted.

### Atomic force microscopy (AFM) of type I collagen fibrils

Type I collagen from rat tail (Discovery Labware Inc., Bedford, MA) was diluted in 10 mM phosphate buffered saline (PBS) pH 7.4 to a final concentration of 2.0 mg/mL and incubated at 37 °C for 2 hours for fibril self-assembly. A sample volume of 20 μL was deposited on a 1 cm × 1 cm square of freshly cleaved mica (Ted Pella Inc., Redding, CA) and incubated at room temperature for 15 min. Then the surface of the sample was washed with 1 mL of deionized water and left to dry at room temperature for 1 h before being imaged. The samples were imaged by an NX-10 instrument (Park Systems, Suwon, South Korea) in non-contact mode with PPP-NCHR tips (nominal force constant 42 N/m; 330 kHz frequency; Nanosensors, Neuchatel, Switzerland). Images were not filtered and minimal processing was conducted using XEI (Park Systems, Suwon, South Korea).

## Electronic supplementary material

Supplementary Information

## Data Availability

All relevant data in support of the findings of this study are available from the corresponding author by reasonable request.
