# Supplementary material for: Cryptic binding sites become accessible through surface reconstruction of the type I collagen fibril
Source: Sci Rep. 2018 Nov 9;8:16646. doi: 10.1038/s41598-018-34616-z (PMC6226522; doi:10.1038/s41598-018-34616-z)
Supplement: Supplementary file 1 — Supplementary Information [file 41598_2018_34616_MOESM1_ESM.pdf]

Supplementary Information

Cryptic Binding Sites become accessible through Surface Reconstruction of the Type I Collagen Fibril

Jie Zhu, Cody L. Hoop, David A. Case, Jean Baum*

Department of Chemistry and Chemical Biology, Rutgers University, Piscataway, New Jersey 08854, United States

* Correspondence to: jean.baum@rutgers.edu

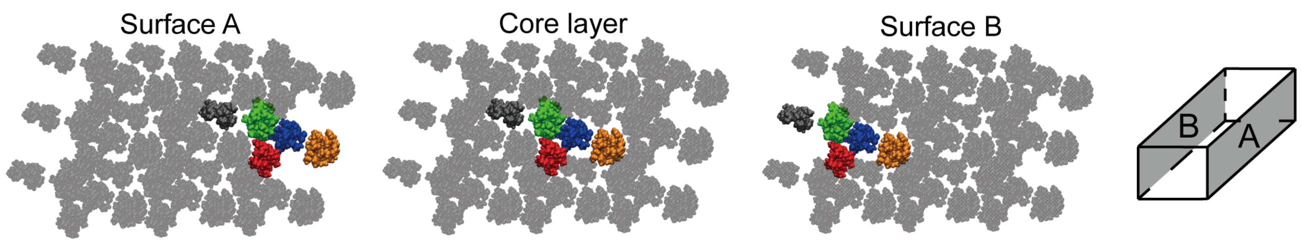

Figure S1. The three SRUs analyzed in the MD simulations to represent surface A, B and core layer are highlighted in the cross-sectional view of collagen fibril model. The box shows the orientation of the fibril surfaces, shaded in gray, relative to the model.

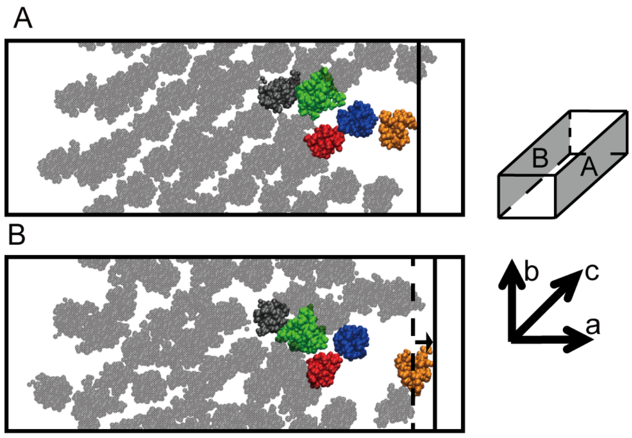

Figure S2. Cross-sectional views of slices taken at 10 to 12 nm from the N-terminus along the longitudinal axis (c-axis) of the collagen fibril model. The surface layer of the overlap region, highlighted by colors, expands outward (B) compared to the starting structure (A). Solid black lines show boundaries of the surface layers and dashed black line shows the original position of the layer boundary.

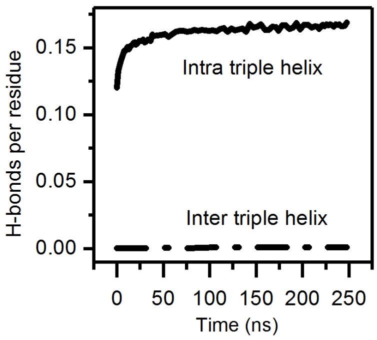

Figure S3. Buildup of backbone–backbone intra- (solid) and inter-triple helix (dashed) protein–protein H-bonds per residue in the 3a3b collagen fibril model through time.
